# Sphenoidal artery: review of the literature and analysis of a dissected arterially injected fetal orbit

**DOI:** 10.1007/s00276-020-02663-9

**Published:** 2021-01-22

**Authors:** E. Leon Kier, Amit Mahajan, Gerald J. Conlogue

**Affiliations:** 1 Yale University School of Medicine, PO Box 208042, New Haven, CT 06520-8042 USA; 2 Quinnipiac University, 275 Mount Carmel Ave, Hamden, CT 06518 USA

**Keywords:** Sphenoidal artery, Middle meningeal artery, Ophthalmic artery, orbit, Superior orbital fissure, Hyrtl canal

## Abstract

**Purpose:**

The sphenoidal artery is considered a component of the complex and dangerous arterial anastomoses of the human orbitocranial region, particularly with the advent of interventional neuroimaging. The objective of this publication was to analyze the various descriptions of the sphenoidal artery in the literature as related to relevant photographs of a dissected arterially injected fetal middle cranial fossa and orbit.

**Methods:**

Publications dealing with middle meningeal-ophthalmic arterial anastomoses, focusing on the sphenoidal artery, were reviewed. A relevant dissection of a fetal specimen was analyzed.

**Results:**

The literature dealing with the sphenoidal artery is at times not in agreement. The nomenclature and anatomy of its passage through the superior orbital fissure or Hyrtl canal have variable descriptions. Photographs of the skull base of a dissected arterially injected fetal specimen show bilateral prominent orbital branches of the middle meningeal arteries. These branches entered both orbits in a course similar to the diagrammatic representations of the sphenoidal artery, and give rise to several major intraorbital arteries. This study provides the only photographic image in the literature of this variation in a human fetal anatomic dissection.

**Conclusions:**

Review of the literature dealing with the sphenoidal artery shows inconsistent nomenclature and conflicting descriptions of its anastomotic connections, and varying evolutionary and embryologic theories. Analysis of the dissected fetal skull base indicates that the sphenoidal artery is not a distinct artery but just a middle meningeal orbital arterial branch, an important component of the complex and dangerous arterial anastomoses of the human orbitocranial region.

## Introduction

The sphenoidal artery (SA) is considered a component of the complex and dangerous arterial anastomoses of the human orbitocranial region, particularly with the advent of interventional neuroimaging. Superselective angiography has enlarged the knowledge of the complex anatomic and functional relationships in the orbitocranial region between branches of the ophthalmic (OA) and middle meningeal (MMA) arterial branches, highlighting potential dangerous complication of intra-arterial therapeutic embolization, such as blindness or stroke. An issue in analyzing the orbital evolutionary, embryologic and adult vascular anatomy literature has been the difficulty in conceptualizing certain nomenclatures such as the recurrent lacrimal and recurrent meningeal arteries (RMA) as well as the introduction of names such as the SA.

The term SA likely was first used in 1971 [4]. Although the *SA* has been pointed out on a number of published cerebral angiograms, its cerebral angiographic anatomy is not validated by photographs of anatomic dissections correlated with angiographic findings. The SA is described by the occasional use of schematic representations or in partially visualized dissections without accompanying neuroimaging.

Additional problems with the literature dealing with the anatomy of the SA are the conflicting descriptions of its anastomotic connections, its passage through the superior orbital fissure (SOF) or Hyrtl canal (HC). Schematic analyses of the SA variations have been published, postulating that it may represent a neomorphic mutation, appearing first in hominoid cranial circulation, and that it may not be a remnant of the stapedial arterial system.

## Materials and methods

### Analysis of publications

The objectives were to review the articles dealing with middle meningeal-ophthalmic arterial anastomoses, focusing on the SA. A large number of articles that deal with the orbital arterial supply, but which do not discuss the sphenoidal artery, were reviewed and excluded from this investigation.

The articles dealing with the SA were analyzed as regards to its definition, how it enters the orbit, the consistency of its descriptions, as well as the presence of diagrammatic representations, cerebral angiography, and anatomic dissections. In addition, a previously unpublished dissection of an arterially injected fetal specimen was analyzed, in view of its relevance to the analysis of the SA.

### Examination of fetal material

During the years 1970–1973, fetal specimens were obtained as part of the material for studies of the evolutionary and embryologic changes of the central nervous system, skull and spine. Some of these specimens were injected intra-arterially with a radiopaque barium–gelatin contrast agent.

Arterial injections were performed manually under fluoroscopic control, by inserting a polyethylene catheter into the umbilical artery of the specimens. Following injection, fixation of the gelatin component of the contrast media was achieved by immersing the specimens in 10% buffered formalin prior to dissection, which were performed using a magnifying lamp and a dissecting microscope.

## Results

### Dissections of specimen

**Fig. 1:**
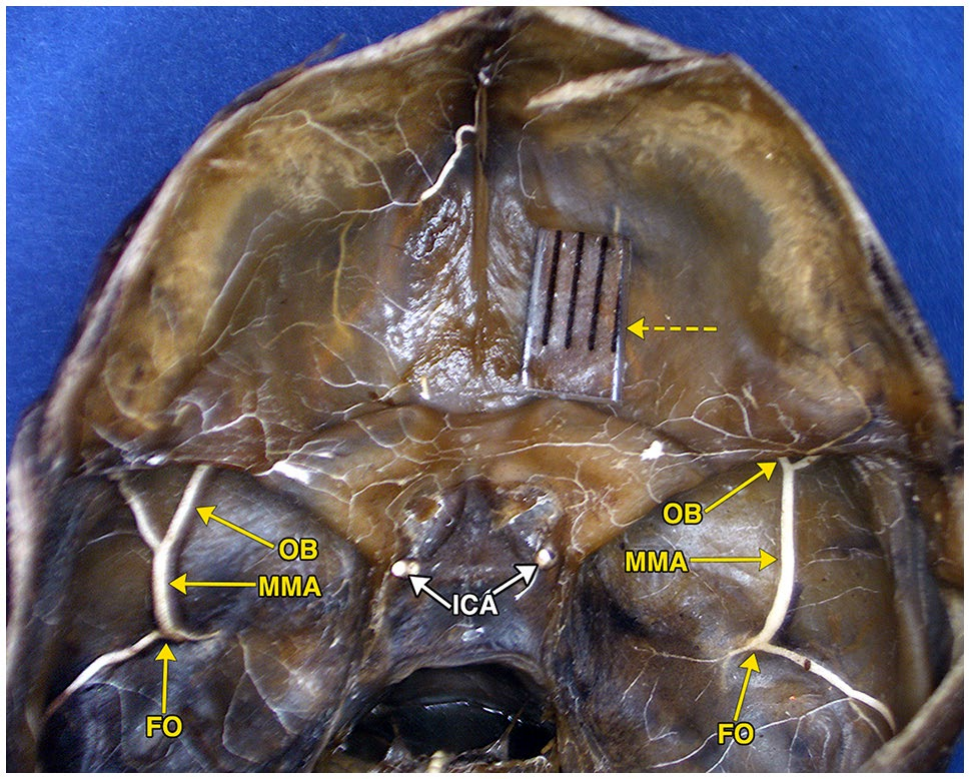
Photograph of the skull base of an 18 weeks’ gestational age specimen, with a crown–rump length of 150 mm and occipito-frontal diameter of 50 mm. The dissection demonstrates the anterior branch of the middle meningeal artery (MMA), injected with a barium–gelatin contrast agent, exiting the foramen ovale (FO) in both middle fossae. The anterior branches divide into the orbital branches (OB) which pass anteriorly to disappear below the posterior edge of the lesser wing of the sphenoid bone. A dashed arrow points to a segment of transparent ruler. The distance between two black lines on the ruler is 1 mm

**Fig. 2:**
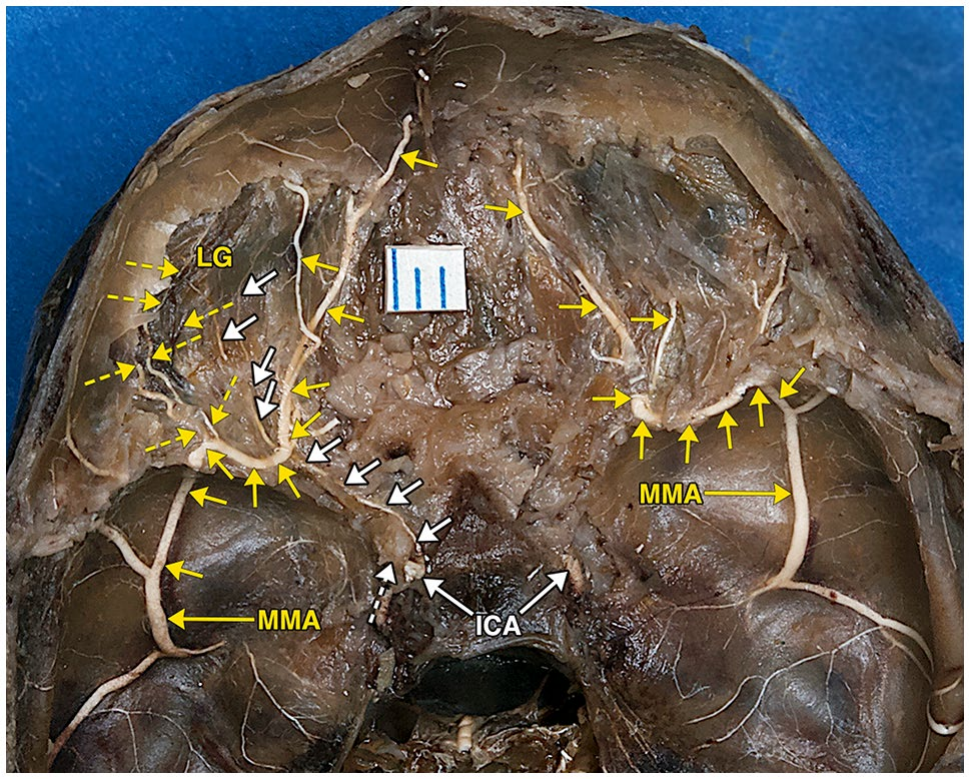
Photograph of the same specimen as in in Fig. 1. Following further dissection of the partially ossified sphenoid bone and removal of the orbital roofs, the prominent orbital branches of the middle meningeal arteries (yellow short arrows) course though the dissected sphenoid bones and enter both orbits, in a course similar to the diagrammatic representations of the so-called sphenoidal artery. The middle meningeal orbital branches divide within the orbit. On the left side, following removal of the roof of the optic canal, an extremely thin ophthalmic artery (white short arrows) originating from the internal carotid artery (ICA), passes underneath the main orbital middle meningeal branch towards the region of the left lacrimal gland (LG). Also visible are a number of small branches (dashed small yellow arrows) originating from the intra-sphenoid middle meningeal orbital artery, coursing towards the region of the lacrimal gland (LG). No ophthalmic artery was identified on the right side. The dashes white arrow points to the dissected anterior clinoid. As the sphenoid bone is partially ossified at this developmental stage, the SOF and HC could not be identified. The distance between two blue lines on the white ruler segment is 1 mm, indicating the small size of some of the visualized vasculature

Photographs (Figs. 1, 2) of a dissected fetal specimen skull base showed prominent middle meningeal orbital arterial branches, passing through the anterior sphenoidal wall of both cranial fossae**,** entering both orbits.

### Description of publications

Likely the earliest mention of the SA was in an article published in 1971 [4]. This article was not reviewed as no copy could be obtained.

Vignaud et al. [33] in 1972 states “we must stress the importance of the sphenoidal artery which links the middle meningeal and lacrimal arteries. This branch crosses the sphenoidal fissure”. On a single lateral radiograph of a common carotid cerebral angiogram, this article points to a small short arterial segment near the MMA, that the authors name the SA. This radiograph was not accompanied by a diagram. As none of the references listed in this article were cited within the article, it is not clear if the SA name came from a previous publication.

A 1974 article by Vignaud et al. [34] states that “The lacrimal artery sometimes originates from the middle meningeal artery via the sphenoidal branch instead of from the ophthalmic artery”. This article cites the previous 1972 article [33] and does not include diagrams or cerebral angiograms depicting the SA.

A 1975 article by Lasjaunias et al. [18] describes the SA as visible during selective external carotid artery injections, originating from the MMA anteriorly in the middle cranial fossa having 2 bends before entering the orbit either through the SOF or HC. Parts of it are difficult to visualize on angiography because of its proximity to bone.

The frequently cited 1975 article by Lasjaunias et al. [17] discussing the SA, includes the following statements:

1. “the recurrent meningeal … and the meningolacrimal artery (less preferably named the sphenoidal artery}”
2. “the recurrent meningeal artery connects the lacrimal artery with the anterior branch of the middle meningeal artery via the superior orbital fissure.”
3. “The Meningolacrimal Artery (Sphenoidal Artery)”
4. “the meningolacrimal artery before it reaches the lesser wing of the sphenoid bone. It then runs transversely and enters the orbit through the canal of Hyrtl (meningolacrimal foramen)”.

This frequently cited article does not cite any references relating to the SA or cerebral angiograms depicting the SA. This article mentions several times that the meningolacrimal (sphenoidal artery) supplies a portion or of the lacrimal territory. The articles by Lasjaunias et al. [20] and Merland et al. [23] schematically show a “sphenoidal branch” of the MMA which runs along the lesser wing of the sphenoid bone, taking part in the dural vascularization of the superior surface of the lesser wing of the sphenoid and middle fossa. The legend of one of these schematic drawings notes that the sphenoidal branch of the MMA is anastomotic with the lacrimal artery (LA) [20]. It is unclear if the “sphenoidal branch” discussed in these articles is the same as the SA.

It is of interest that in view of the frequent citations and referencing of the article by Lasjaunias et al. [17], the term SA does not reappear in any of the subsequent articles and books authored or coauthored by Lasjaunias. The term SA is not used by Hayreh and Dass [15] or by Dorcas Padget in her extensive study of the arterial development of the human embryo.

Ducasse et al. [6] note that when there is persistence of the middle segment of the supra orbital branch of the stapedial artery, it will result in the formation of “an anastomosis between the LA and MMA arteries which is referred to by many authors as the sphenoidal artery”, A figure legend in this article states “an anastomotic meningolacrimal ramus, referred to as the sphenoidal artery”*.* This article includes a photograph of a dissection showing a very short segment of the “Sphenoidal artery” originating from the MMA and entering the orbit, apparently though HC. In another article, Ducasse et al. [7] mention, in the French summary, that the SA is another name for the meningolacrimal (MLA) anastomotic branch, This article includes a photograph of a dissection showing a very short segment of the “Sphenoidal artery” originating from the LA. Thus, both articles follow the Lasjaunias et al. [17] definition.

Gregg et al. [12], similarly to Lasjaunias et al. [17] definition, note that “the meningolacrimal or sphenoidal artery” may become a collateral supply from the MMA for the ICA or OA in cases of ICA occlusion.

Diamond [5] significantly expanded the discussion of the SA. When quoting the Lasjaunias et al. 1975 article [17], Diamond’s article changed the definition of the SA, In the 1975 Lasjaunias article[17], Diamond’s publication stated that “…… henceforth referred to as the sphenoidal artery again after Lasjaunias et al. (1975), who also referred to it as the ‘recurrent meningeal artery”. The above quotation is different from the description in the 1975 Lasjaunias et al. [17] article.

This changed definition is also used by Gailloud et al. [10] that extensively depicts the *SA* in the text, as well as schematic representations, and detailed high-resolution superselective cerebral angiograms. It again cites the 1975 Lasjaunias et al. [17] article and states “one connecting branch is short and straight; it crosses the foramen of Hyrtl (or cranio-orbital foramen) and takes the name, meningolacrimal artery. The other is long and tortuous; passes through the superior orbital fissure (SOF) and takes the name sphenoidal artery.” In the text and legends of some of the schematic representations, the MLA and the SA are mentioned as separate arteries.

Macchi et al. [21] in describing the anatomy of the canals connecting the orbit with the cranial cavity note that in cases where the embryonic stapedial artery gives off the lacrimal artery in the cranial cavity, this results in direct communication between the MMA and the OA through the SOF which in the adult “is referred to as the meningo-ophthalmic or sphenoidal artery”.

Bonasia et al. [1] in a review of the stapedial artery note that in the embryologic development of the inferolateral trunk of the stapedial artery… “the lacrimal artery (sphenoidal artery}, which penetrates the orbit through the superior orbital fissure.” The article also includes a superselective angiographic figure of an OA arising from the MMA, via the “sphenoidal artery”. Later in the same article the authors state that “the medial branch (referring to the stapedial artery) passing through the superior orbital fissure is named the recurrent meningeal artery in its intraorbital segment and sphenoidal artery in its intracranial segment.”

In an accompanying review of the MMA, Bonasia et al. [2] in a diagrammatic representation of the anastomoses of the MMA, depict the SA and the meningolacrimal artery as communicating with the lacrimal artery. In a table listing the branches of the MMA, the SA is described as possibly anastomosing with the recurrent meningeal branch of the OA and the inferolateral trunk of the ICA.

Several highly detailed dissections of the vascularity of adult cadaveric orbital specimens have been published [8, 22, 26, 27]. None of these published dissections outline the entire SA or include cerebral angiograms.

Martins et al. [22] list the SA as a branch of both the LA and the medial branch of the anterior division of the MMA. The authors note that the “RMA (sphenoidal)” runs in the sphenoparietal sulcus with the sphenoparietal sinus. The presence of both the MLA and the “RMA-or SA” is identified in the same orbital dissection, noting dual connection between the MMA and the LA. The authors identify the MLA and the tortuous RMA and note that the former passes through the lacrimal canal (HC) and the latter through the SOF.

Perrini et al. [26] state “This branch originating from the MMA has been referred to as a RMA, orbital branch, or sphenoidal artery of the MMA”.

Pretterklieber and Krammer [27] describe a dissected adult specimen with an absent foramen spinosum and the MMA was replaced by the “sphenoidal artery” a branch of the OA. The “sphenoidal artery” enters the middle cranial fossa through the SOF where it replaced the MMA. The authors name this artery as the ophthalmic-meningeal artery (OMM).

Erdogmus and Govsa [8] citing Diamond [5] but not Lasjaunias et al. [17], in a dissection study of the arterial supply of the lacrimal gland note that the RMA which is also called the SA passes through the SOF while the other MMA branch is known as the meningo-orbital or MLA passes through the cranio-orbital foramen. These authors refer to the *SA* as the anastomotic artery. This likely is the same artery named the ramus anastomoticus cum arteria lacrimali.

Konishi and Kikuchi [16] analyzed variations of the ramus anastomoticus cum a. lacrimali in 300 orbits. Konishi and Kikuchi article [16] includes photographs of a detailed dissection demonstrating the ICA, OA, LA and the ramus anastomoticus cum a. lacrimali. It is not clear from the review if the ramus enters the orbit through the SOF or HC.

## Discussion

In view of the importance of the SOF and HC for the issues discussed in this study, these structures will be described first.

The SOF is listed in the 1998 Terminologia Anatomica [26]. The oculomotor, trochlear, abducens, lacrimal, nasociliary, and frontal nerves traverse the SOF. Lasjaunias et al. [19] and Willinsky et al. [35] describe the deep recurrent OA passing through the SOF to reach the cavernous region. Moret et al. [24] note that the recurrent meningeal artery also traverses the SOF.

Hyrtl canal or foramen (HC), a small opening within the greater wing of the sphenoid, at varying positions lateral to the SOF, has been described under various names. HC has been used [25, 26]. It has also been named the cranio-orbital foramen [9, 36] meningo-orbital foramen [11, 29, 30], lacrimal foramen [17, 21], and orbitomeningeal foramen [21, 28]. HC and its other names are not listed in the 1998 Terminologia Anatomica [31]. HC is the persistence of an embryonic canal for the supraorbital division of the stapedial artery as it enters the orbit and anastomose with the lacrimal branch of the OA [9].

### The sphenoidal artery

Sphenoidal artery is the name used, to describe an arterial structure which originates from the middle meningeal artery and connects with the lacrimal artery in the orbit, supplying the lacrimal gland region. Review of the literature dealing with the SA shows inconsistent nomenclature and descriptions of its anastomotic connections. These disagreements focus on the course and nomenclature of the arterial structure connecting the MMA with the LA, which either traverses through the superior orbital fissure or through HC, and rarely through both.

Vignaud et al. [33] describe a vessel passing through the sphenoidal foramen, connecting the middle meningeal artery to the lacrimal artery while Lasjaunias et al. [17] consider it the same as the meningolacrimal artery (MLA) passing through the cranio-orbital foramen/Hyrtl canal and described a separate RMA, passing through the SOF. Ducasse et al. [6] use the same terminology as Lasjaunias et al. [17].

Diamond [5] quoting Lasjaunias et al. 1975 article [17] changed the description of the SA in that article [17] as describing the SA passing through the SOF, not through HC. His quotation from Lasjaunias et al. 1975 article is different from the description in the 1975 Lasjaunias et al. article [17].

Diamond’s [5] changed definition is also used by Gailloud et al. [10], describing the SA as passing through the lateral SOF, a RMA through the medial SOF and a MLA through the Cranio-orbital foramen.

Erdogmus and Govsa [8], Martins et al. [22] and Perrini et al. [26] consider it the SA as same as the RMA and describe it as passing through the SOF. Pretterklieber and Krammer [27] describe it as a vessel passing through the SOF and supplying the middle meningeal artery from the OA in the absence of a foramen spinosum.

In discussing the meningo-orbital foramen (HC), Georgiou and Cassell [11] state that it is an embryonic conduit for the supraorbital division of the stapedial artery en route to the orbit. In reviewing the variation in the formation of the various segments of the orbital circulation, they note that the LA and RMA are not 2 separate arteries. Embryologically, both are the same vessel originating from the supraorbital division of the stapedial artery. The position of the supraorbital division of the stapedial artery will determine if the connection between the anterior division of the MMA and the LA branch of the OA passes through the SOF or the meningo-orbital foramen (HC).

Georgiou and Cassell [11] also address the theory proposed by Diamond [5], and later discussed by Gailloud et al. [10], that the SA may represent a neomorphic mutation, appearing first in hominoid cranial circulation, and that it may not be a remnant of the stapedial artery. These authors take issue with the suggestion of Diamond’s article [5] that the vessel traveling through the SOF is a neomorph, and indicate that it is an unnecessary explanation. The variations in the adult anatomy can be explained by the position and branching pattern of the supraorbital division of the stapedial artery.

Georgiou and Cassell [11] also briefly discuss the evolutionary change of origin of the OA from either the ICA or the external carotid artery and the various intermediate evolutionary stages in this transformation. They mention studies in various animals with varying sized contribution from either the ICA or the ECA branches. These evolutionary transformations can explain many of the human anomalies which has been analyzed by many authors. Hayreh[14] notes that in the rhesus monkey, which has a normal OA, the LA has a significant connection with the MMA. Hayreh and Dass in their extensive list of articles, going back to the early nineteenth century, that describe the orbital blood being supplied partially or wholly from the MMA, do not mention the SA [15].

Diamond [5] starts his article, introducing the term SA, by mentioning the ramus anastomoticus cum a. meningea media as an anastomotic ramus of the MMA that enters the SOF to join the LA. As noted by Diamond [5], this anatomic term was used to describe an anastomotic ramus of the anterior branch of the MMA entering the orbit either through the SOF or through HC. Diamond does not indicate that the SA may be just a new name for the ramus anastomoticus cum a. lacrimali.

According to Terminologia Anatomica [31], the appropriate term is ramus anastomoticus cum a. lacrimali. Terminologia Anatomica list two separate terminologies: ramus anastomoticus cum a. meningea media (A12.2.06.028) as a branch of the lacrimal artery; and ramus anastomoticus cum a. lacrimali (A12.2.05.068) as a branch of the maxillary artery.

Toma [32] in his review of the embryology of the OA notes in one of his schematic diagrams that various names such as the SA, meningo-ophthalmic and recurrent meningeal arteries are used to identify the same artery.

The photographs of the dissected fetal specimen (Figs. 1, 2) showed prominent middle meningeal orbital arteries branches, passing through the anterior sphenoidal wall of both cranial fossae**,** entering both orbits in a course similar to diagrammatic representations of the meningolacrimal (sphenoidal) artery by Lasjaunias et al. [17], the meningolacrimal and nasociliary branches of the supraorbital artery by Moret et al. [24], and the SA by Diamond [5] and Gailloud et al. [10].

Through which foramen, the SOF or the HC, the MMA branch in the dissected specimen passed, could not be determined because of the incomplete ossification of the anterior sphenoidal wall at that fetal age. Even in dry adult skull specimens as measured by Yoon and Pather [36] and by Macchi et al. [21], there is close proximity of the cranio-orbital foramen (HC) to the SOF. Yoon and Pather study showed a distance of 8.05 ± 3.40 mm. The study by Macchi et al. showed a distance of 9.62 ± 4.33 mm.

Similar to the major supply of the orbit by the orbital branch of the MMA in the dissected fetal specimen (Figs. 1, 2) are two adult case descriptions in the literature. Chanmugam [3] describes diagrammatically a case in which the ICA supplies the globe and optic nerve, and the MMA supplies the lacrimal gland and all the other orbital structures. Harvey and Howard [13] describe diagrammatically the entire orbital circulation arising from the MMA.

## Conclusions

Review of the literature dealing with the MMA–LA anastomoses demonstrated various descriptions, evolutionary, and embryologic theories for these anastomoses. Some authors have different names for the same vessel. The sphenoidal artery literature shows inconsistent nomenclature and descriptions of its course and anastomotic connections. It appears that the SA is just an additional name for the ramus anastomoticus cum a. lacrimali, possibly a factor why the so-called sphenoidal artery is not listed in Terminologia Anatomica. Analysis of the dissected fetal skull base confirms that the SA is not a distinct artery, just a middle meningeal arterial orbital anastomotic branch, an important component of the complex and dangerous arterial anastomoses of the human orbitocranial region.
